# A comparative transcriptomic analysis reveals a coordinated mechanism activated in response to cold acclimation in common vetch (*Vicia* *sativa* L.)

**DOI:** 10.1186/s12864-022-09039-w

**Published:** 2022-12-08

**Authors:** Rui Dong, Ben Luo, Li Tang, Qiu-xia Wang, Zhong-Jie Lu, Chao Chen, Feng Yang, Song Wang, Jin He

**Affiliations:** 1 Department of Grassland Science, College of Animal Science, Guizhou University, Guiyang, China; 2 Key Laboratory of Animal Genetics, Breeding and Reproduction in the Plateau Mountainous Region, Ministry of Education, Guizhou University, Guiyang, China; 3 School of Tropical Crops, Hainan University, Haikou, China; 4 State Key Laboratory of Grassland Agro-ecosystems, China, College of Pastoral Agriculture Science and Technology, Lanzhou University, Lanzhou, China; 5 Grassland Technology Experiment and Extension Station, Guiyang, China; 6 College of Agriculture, Guizhou University, Guiyang, China

**Keywords:** Ommon vetch, Cold acclimation, Transcriptome analysis, Weighted correlation network analysis (WGCNA), Cold response

## Abstract

**Background:**

Due to its strong abiotic stress tolerance, common vetch is widely cultivated as a green manure and forage crop in grass and crop rotation systems. The comprehensive molecular mechanisms activated in common vetch during cold adaptation remain unknown.

**Results:**

We investigated physiological responses and transcriptome profiles of cold-sensitive (Lanjian No. 1) and cold-tolerant (Lanjian No. 3) cultivars during cold acclimation to explore the molecular mechanisms of cold acclimation. In total, 2681 and 2352 differentially expressed genes (DEGs) were identified in Lanjian No. 1 and Lanjian No. 3, respectively; 7532 DEGs were identified in both lines. DEGs involved in “plant hormone signal transduction” were significantly enriched during cold treatment, and 115 DEGs involved in cold-processed hormone signal transduction were identified. Common vetch increased the level of indoleacetic acid (IAA) by upregulating the transcriptional regulator Aux/IAA and downregulating GH3, endowing it with stronger cold tolerance. An auxin-related DEG was overexpressed in yeast and shown to possess a biological function conferring cold tolerance.

**Conclusion:**

This study identifies specific genes involved in Ca^2+^ signaling, redox regulation, circadian clock, plant hormones, and transcription factors whose transcriptional differentiation during cold acclimation may improve cold tolerance and contributes to the understanding of common and unique molecular mechanisms of cold acclimation in common vetch. The candidate genes identified here also provide valuable resources for further functional genomic and breeding studies.

**Supplementary Information:**

The online version contains supplementary material available at 10.1186/s12864-022-09039-w.

## Introduction

In nature, low temperature, a variable environmental pressure and one of the main abiotic stresses, substantially limits the growth and development, geographic distribution and productivity of plants [1]. As a common natural disaster in high-elevation and temperate regions, cold injury potentially causes a series of symptoms in crops, including stunted buds, leaf wilting/yellowing, reduced tillering and tissue death [2]. Overall, cold injury causes a loss of approximately $2 billion in global agricultural productivity each year, and approximately 13 million hectares of rice (*Oryza sativa* L.) alone are affected by low temperatures each year [1, 3]. Plants have evolved various strategies through natural selection to cope with cold stress that enable their survival. Cold acclimation is one of the key mechanisms by which plants adapt to low-temperature stress [4].

Most plants adapted to temperate and northern climate zones are cold-tolerant. However, some species undergo cold acclimation, with changes in transcription, biochemistry and morphology, which may improve resistance to frost [5]. This process is achieved by exposing plants to low temperatures greater than 0 °C for a period of time (usually several days or weeks) [2]. The increase in reactive oxygen species (ROS) levels, upregulation of antioxidant enzymes, changes in plant hormones, and increases in levels of metabolites such as soluble sugars are all biochemical changes related to cold acclimation in plants [6]. Therefore, identifying and characterizing the strategies controlling the cold domestication of plants are keys to maintaining crop productivity in crop improvement programs aimed at solving serious food shortages in the future [6, 7].

Gene expression in response to cold stress in plants typically involves posttranslational histone modifications, including acetylation, methylation, and phosphorylation [8]. Cold regulation (COR) genes play a role in plant responses to low-temperature stress, and some *COR* genes in *Arabidopsis* encode key enzymes required for the synthesis of osmotic adjustment substances [9]. These enzymes increase the frost tolerance of plants by producing and increasing the accumulation of cryoprotective proteins, soluble sugars and other metabolites, thereby repairing the cold-rigidified membrane and stabilizing the cell osmotic potential [10]. In *Arabidopsis*, the WD40 repeat protein HOS15 is involved in histone deacetylation and cold tolerance. Histone deacetylase 2C (HD2C) interacts with HOS15, and both proteins interact with the promoters of the cold-responsive genes *COR15A* and *COR47*. HOS15 interferes with the interaction between the *COR* gene promoter and HD2C, which is related to the acetylation of histone H3 [8]. Cold acclimation activates two classical regulatory networks: C-repeat/dehydration-responsive element binding (DREB) factor (CBF)-dependent and CBF-independent transcription pathways [1]. Approximately 12% of the cold-responsive transcriptome is regulated by CBF transcription factors [11]. Three CBF family members have been identified in *Arabidopsis*, namely, CBF1, CBF2 and CBF3 [12]. The CBF protein recognizes the *CRT/DRE* cis element, which contains the conserved CCGAC sequence and is present in the promoter of the *COR* gene subset [10, 13]. In addition, HOS15 interacts with CBF transcription factors to regulate cold induction by binding to the *COR* gene promoter [8]. Low temperature and intense light potentially induce chronic photoinhibition of PSII and the accumulation of ROS, which may lead to photooxidative damage [5, 14].

Previous studies have shown that Ca^2+^ channels are involved in the early cold stress response of higher plants. Low temperature rapidly increases the cytosolic free calcium concentration in *Arabidopsis* and activates calcium permeation channels in mesophyll cells [15]. By blocking Ca^2+^ channels, antagonizing the action of calmodulin, or inhibiting protein kinases through chemical treatment, the frost tolerance of alfalfa (*Medicago sativa* L.) cells is significantly reduced [16]. At 25 °C, the use of chemical reagents that promote calcium influx induces the overexpression of cold acclimation-specific (CAS) genes in alfalfa.

Although the mechanism of cold acclimation has been comprehensively studied in model plants and crops such as *Arabidopsis* and wheat (*Triticum aestivum* L.), relatively few studies on cold acclimation in legumes have been conducted [1, 9]. Common vetch (*Vicia sativa* L.) is an important annual self-pollinated legume forage. Its excellent cold resistance makes it very suitable for planting in alpine environments [1, 17]. Common vetch is an important green manure and fodder crop used in grass field rotation in alpine environments and has been used to produce methane biofuel and health food [18–20]. Turkey has the largest planting area of *V. sativa*, at 579,684 ha, which is larger than that of other annual feed legumes [21]. However, an understanding of the complex gene expression patterns and regulatory mechanism in the cold adaptation process of common vetch is lacking, hindering the study of the genetic and molecular mechanisms of cold stress in common vetch and breeding of cold-tolerant varieties. In this study, common vetch was treated with cold stimulus (CS), chilling acclimation (CA), frost acclimation (FA), and deacclimation (DA) to reveal the key transcription pathways involved in cold stimulation and cold acclimation and to provide novel insights into the regulation of cold-related genes.

## Results

### Cold tolerance evaluation

**Fig. 1:**
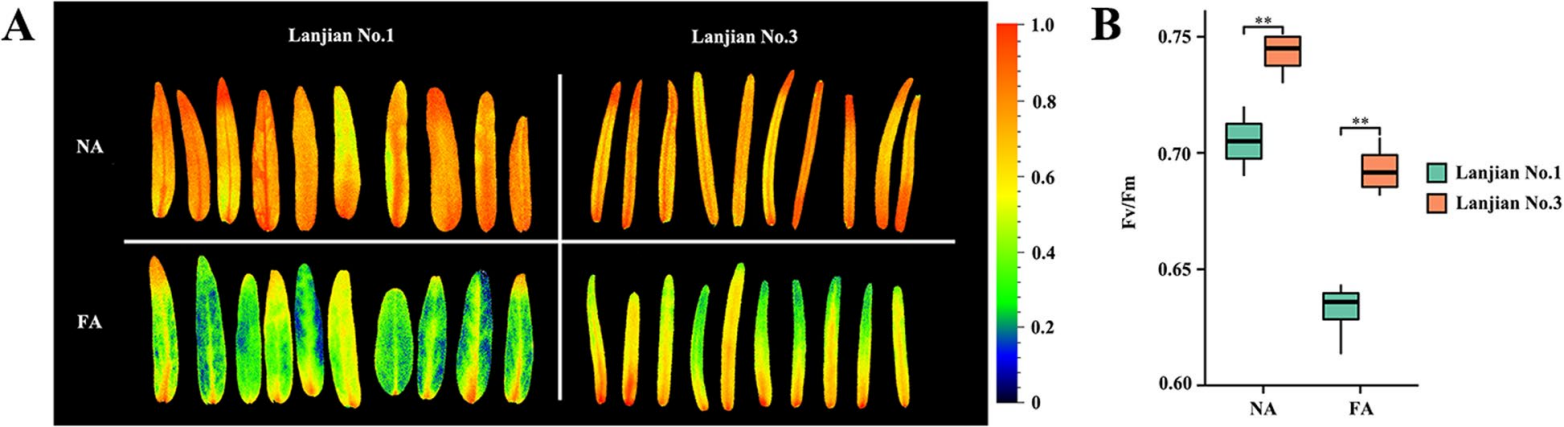
Effects of cold acclimation on chlorophyll fluorescence in two common vetch cultivars. **A** chlorophyll fluore by Fv/Fm image; **B** Fv/Fm value. ** *p* < 0.01

**Fig. 2:**
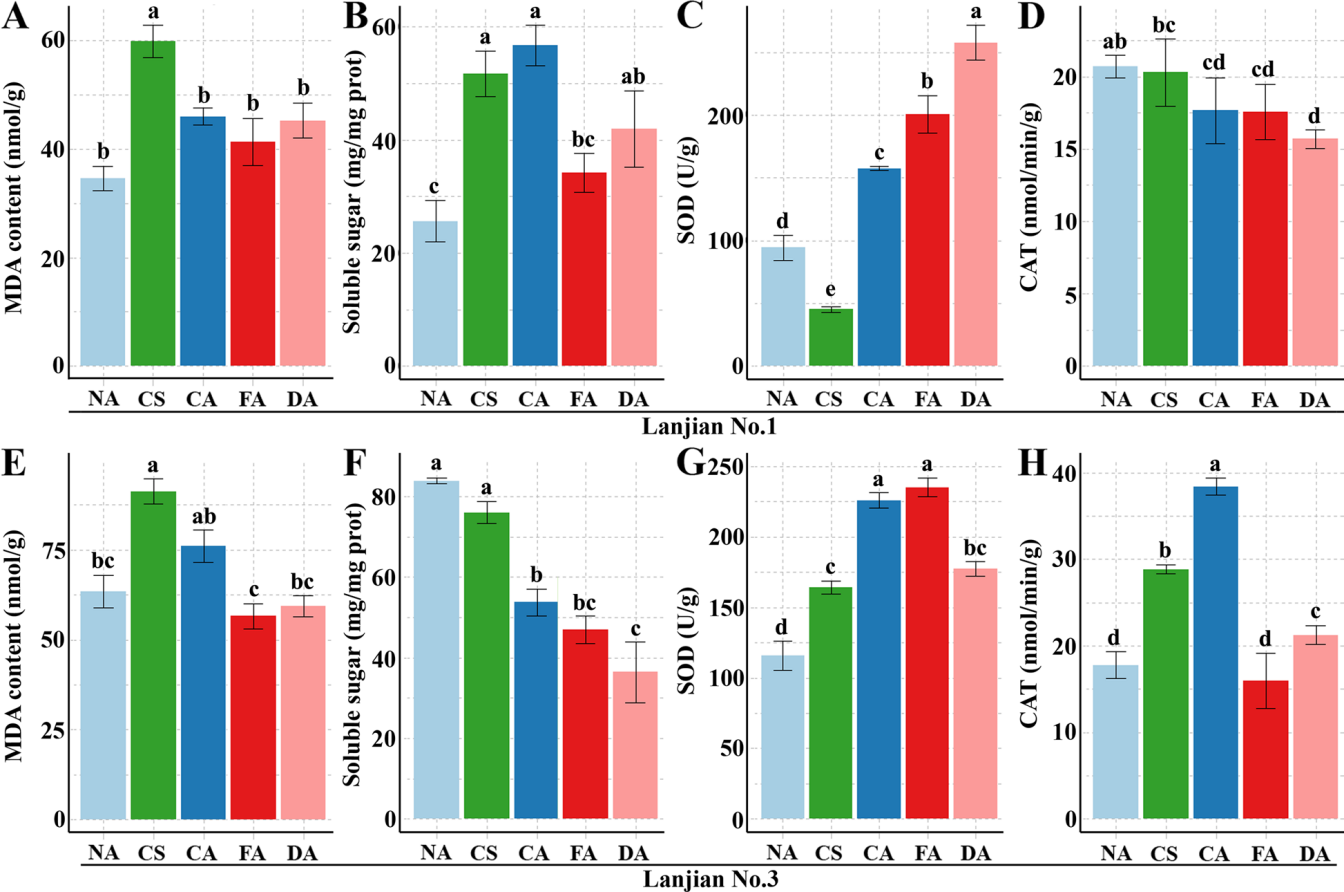
Dynamic physiological changes under different cold treatments. MAD: malondialdehyde; POD: superoxide dismutase; CAT: catalase. *p* < 0.05

We measured the chlorophyll fluorescence in Lanjian No. 1 and Lanjian No. 3 after cold treatment and assessed malondialdehyde (MDA), superoxide dismutase (SOD), catalase (CAT) and soluble sugar contents under different treatment conditions to study the cold tolerance of the two cultivars. After 12 h of FA (4 °C for 5 days) treatment, the chlorophyll fluorescence (FV/FM) values of the two cultivars were lower than those of NA (nondomesticated controls); the value of Lanjian No. 3 was extremely significantly higher than that of Lanjian No. 1 under both treatment conditions (*P* < 0.01) (Fig. 1A–B). The MDA content showed a similar pattern in Lanjian No. 1 and Lanjian No. 3, increasing significantly following CS (4 °C for 6 h) treatment but gradually decreasing upon CA (10 °C for 5 days) and FA treatments (Fig. 2a, e). The soluble sugar content in Lanjian No. 1 increased significantly after CS and CA treatment and decreased significantly in the later stage (Fig. 2b). The soluble sugar content in Lanjian No. 3 gradually decreased after treatment (Fig. 2F). We also assessed the contents of two key antioxidant enzymes, SOD and CAT, to explore the effect of cold stress on the oxygen metabolism system in plants receiving different treatments. The SOD contents in Lanjian No. 1 and Lanjian No. 3 measured after CA and FA treatments were significantly higher than those detected after NA and CS treatments (Fig. 2C, G). The trend for CAT levels in the two cultivars was not the same. Lanjian No. 1 exhibited a decreasing trend in response to cold treatments (Fig. 2D). Lanjian No. 3 had a significantly higher CAT content after CA and FA treatment than after CS treatment (Fig. 2H). The MDA, soluble sugar, SOD and CAT contents in Lanjian No. 3 were significantly higher than those in Lanjian No. 1 after exposure to the CS and CA treatments. A comprehensive evaluation showed that Lanjian No. 1 and Lanjian No. 3 were cold-sensitive and cold-tolerant, respectively, and the two cultivars employed different mechanisms to cope with cold stress and domestication.

### Illumina sequencing and mapping under cold acclimation

**Fig. 3:**
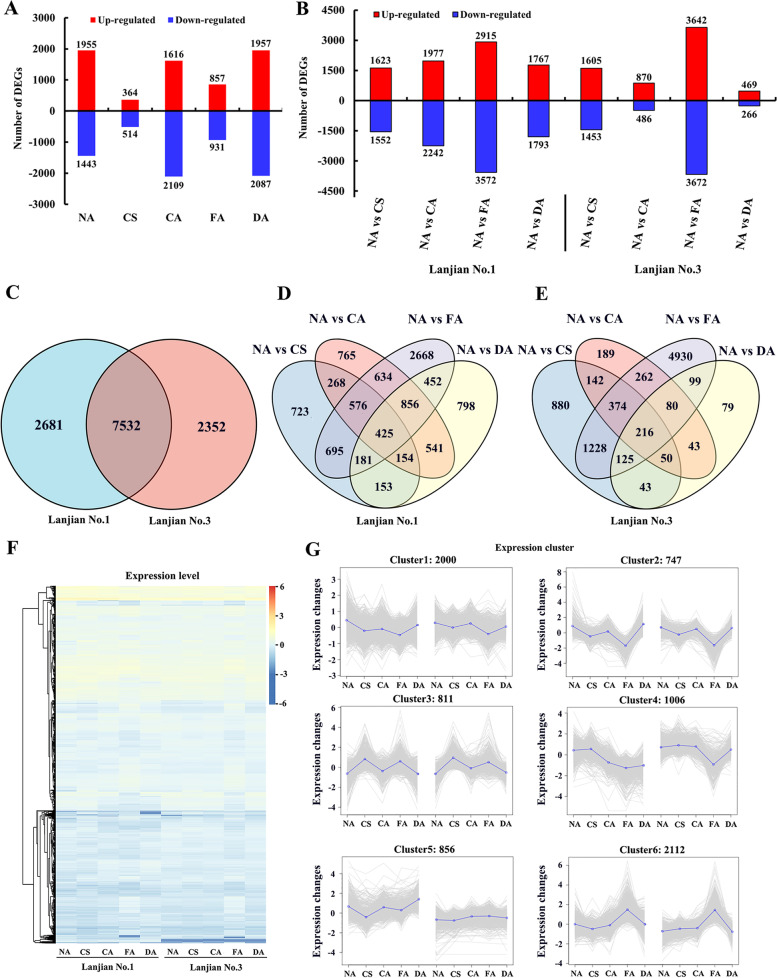
Expression of DEGs during different cold treatments. **A** Number of DEGs in each treatment. **B** Number of DEGs in each treatment for different varieties. **C** Venn diagram showing the distribution of DEGs between two varieties. **D** and **E** Venn diagram showing the distribution of DEGs among the different treatments of two cultivars. **F** Expression level of coexpressed DEGs among cultivars. **G** Expression pattern clusters of DEGs

We performed an RNA-Seq analysis to determine the transcriptome profile in common vetch and understand changes in gene expression during cold acclimation and deacclimation. A total of 12,565 DEGs were generated from 36 RNA samples. Among them, 2681 (Lanjian No. 1) and 2352 (Lanjian No. 3) DEGs were obtained, and the number of DEGs obtained after CA (3725 DEGs) was higher than that obtained after CS (878 DEGs) and FA (1788 DEGs) (Fig. 3A, C). The mean base count of each sample and GC content were 6987.33 M and 42.79%, respectively. Q30 ranged from 94.49 to 95.86%, with an average of 95.20% (Additional file 7 Supplementary Table S4). Ultimately, we obtained average mapped read, unique mapped read, and multimap read ratios of 83.64, 23.41, and 76.59%, respectively (Additional file 7 Supplementary Table S4). The RNA-Seq data have been deposited in the NCBI SRA database (PRJNA820149), and the verifications of the data is shown in Additional file 1 Fig. S1.

### DEGs identified under cold acclimation

Compared with the control treatment NA, CS induced 3175 and 3058 DEGs in Lanjian No. 1 and Lanjian No. 3, respectively, indicating that some genes responded quickly to cold stimulus (Fig. 3B). In the CA treatment group, 4219 and 1356 DEGs were identified in Lanjian No. 1 and Lanjian No. 3, respectively. In general, low-temperature acclimation increased the number of DEGs. However, after FA treatment, 1788 and 7314 DEGs were detected in Lanjian No. 1 and Lanjian No. 3, respectively, indicating that the cold-tolerant cultivar induced the expression of more genes in response to frost and cold resistance after undergoing low temperature acclimation than the cold-sensitive cultivar (Fig. 3B). Among them, 425 and 216 DEGs were coexpressed in Lanjian No. 1 and Lanjian No. 3, respectively, after all treatments (Fig. 3D and E).

The 7532 DEGs that were coexpressed between the two cultivars were divided into 6 main clusters according to their expression patterns (Fig. 3G, F). Among them, cluster 4 (1006 DEGs) and cluster 5 (856 DEGs) showed different expression patterns between the two cultivars, suggesting that these DEGs are candidates contributing to differences in cold resistance. The DEGs in other clusters showed similar patterns in each cultivar.

### Functional comparison of cold-responsive DEGs

**Fig. 4:**
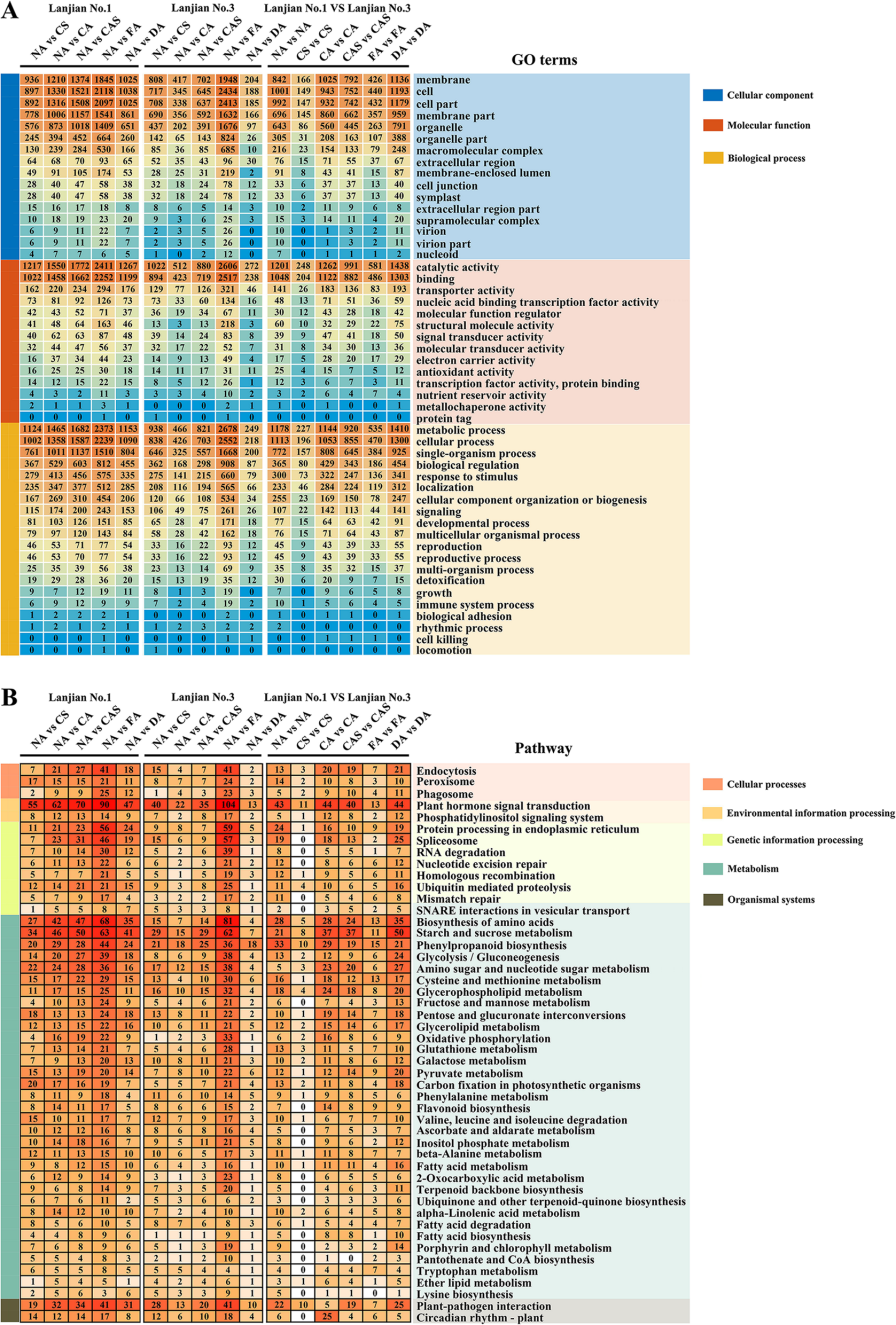
GO and KEGG pathway enrichment analyses. **A** DEGs in each GO term were assigned to cellular components, molecular functions, and biological processes as defined by the GO database. **B** For each treatment comparison, the number of DEGs in each pathway is shown. The distribution of DEGs for each treatment comparison within each cultivar and for each treatment comparison between cultivars was included

We conducted a Gene Ontology (GO) enrichment analysis of different categories to infer the functions of DEGs activated in the two cultivars under cold stress and to determine whether the discovered DEGs are functionally involved in the cold stress response process. We classified all DEGs into the categories of “cellular component”, “molecular function” and “biological process”, with “biological process” identified as the most common category. DEGs showed very similar distributions in enriched GO functional terms in the two cultivars, although the number of DEGs enriched in each GO term differed between the cultivars (Fig. 4A). In the “cellular component” classification, membrane, cell and cell part were the top GO terms. In the “molecular function” category, the top GO terms were catalytic activity, binding and transporter activity. In the “biological process” classification, metabolic process, cellular process and single-organism process were the top GO terms. In addition, the comparisons of the DA vs. DA and CA vs. CA treatments produced the greatest numbers of DEGs between the two cultivars, indicating that the CA treatment may have led to the expression of the most cold stress-responsive DEGs. Simultaneously, Lanjian No. 3 exhibited more DEGs after CA treatment than Lanjian No. 1. This difference may reflect the difference in cold tolerance between the cultivars.

### Kyoto gene and Genome encyclopedia (KEGG) pathways enriched in response to cold

We mapped all DEGs to the KEGG database to retrieve differentially enriched pathways between each cultivar and to understand the characteristics of the complex biological behaviors involved in the cold treatment reaction. Then, we searched the pathways shared by the two cultivars and identified 48 significantly (*P* < 0.05) enriched pathways (Fig. 4B). Among them, “plant hormone signal transduction”, “biosynthesis of amino acids” and “starch and sucrose metabolism” were the pathways enriched in the most abundant DEGs. We also observed different numbers of DEGs enriched in the same pathway between treatment groups in the two cultivars. The most abundant DEGs appeared in the “plant hormone signal transduction” pathway for the comparison of NA with FA, with 90 DEGs identified in Lanjian No. 1 and 104 DEGs identified in Lanjian No. 3. A similar pattern was observed for the “biosynthesis of amino acids” and “starch and sucrose metabolism” pathways. In the comparisons of NA with CS and NA with CA, Lanjian No. 1 displayed more DEGs in all 48 pathways in most cases. Among the three comparisons, the DEGs in Lanjian No. 1 showed an increasing trend, whereas those in Lanjian No. 3 first showed a decreasing and then an increasing trend. Therefore, the cold-sensitive cultivar changed its gene expression pattern earlier to cope with cold stress. However, cold acclimation significantly induced the expression of cold tolerance-related DEGs, and the DEGs that were uniquely expressed in Lanjian No. 3 during this period may be key genes responsible for its cold tolerance.

### Identification of TFs

**Fig. 5:**
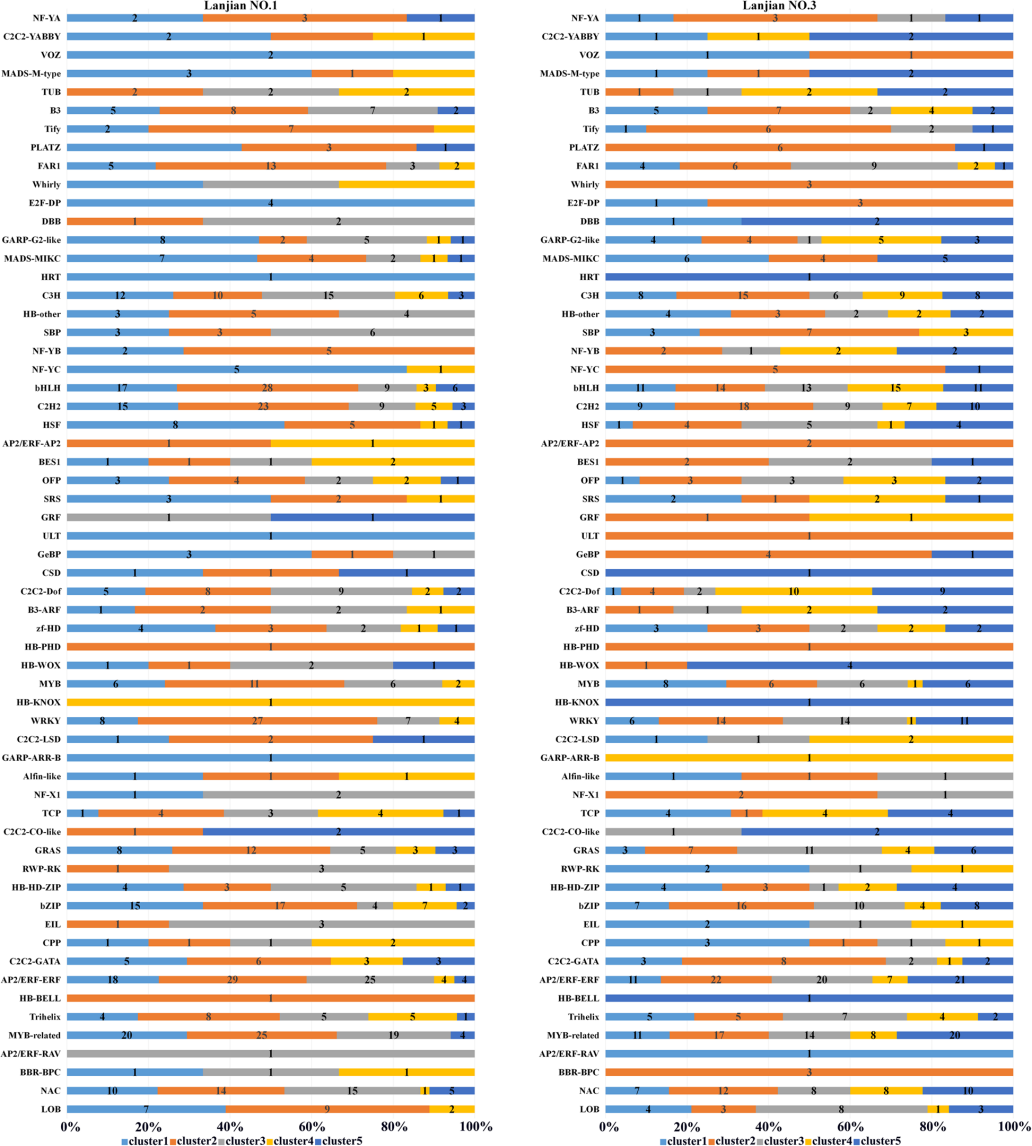
Expression distribution of cold-responsive transcription factor DEGs in the two varieties

Among all DEGs, 60 TF families shared by the two cultivars were identified: 887 DEGs in the Lanjian No. 1 TF family and 889 in the Lanjian No. 3 TF family (Fig. 5). Members of the AP2/ERF-ERF (80 and 81 for Lanjian No. 1 and Lanjian No. 3, respectively) family were the most abundant, followed by the MYB-related (68 and 70), bHLH (63 and 64), C2H2 (55 and 53) and WRKY (46 and 46) families. In addition, we obtained the expression pattern of TFs in each of the two cultivars based on the self-organizing tree algorithm using MEV (Multiple Experiment Viewer) 4.9.0 software (clusters 1–5 for Lanjian No. 1 and Lanjian No. 3) (Fig. 5). Most of the TF families in the two cultivars exhibited a similar expression pattern under cold stress, such as AP2/ERF-ERF, MYB-related, bHLH, C2H2, and WRKY; a few TF families showed different expression patterns between the two cultivars, such as C2C2-Dof, HSF, MADS-MIKC, and TCP.

### Identification of DEGs involved in plant hormone signal transduction

**Fig. 6:**
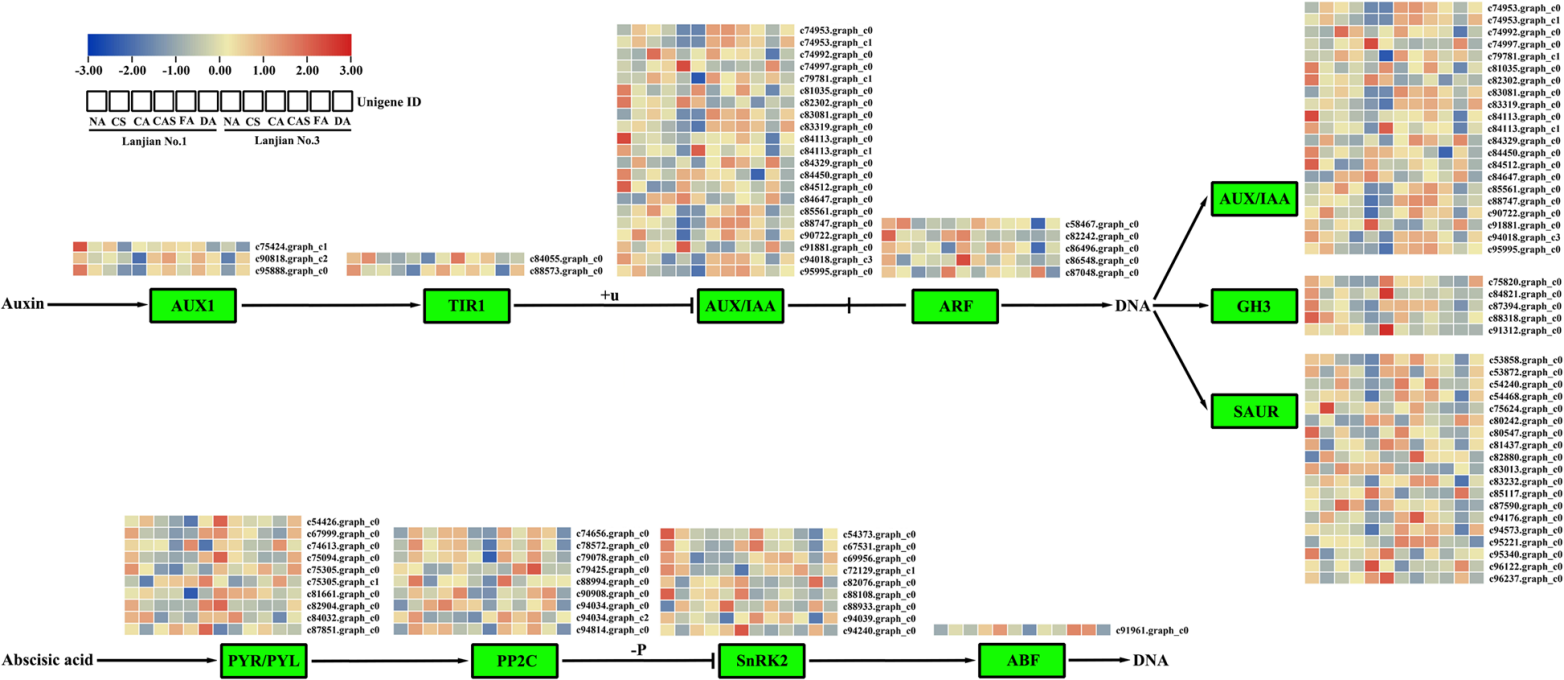
DEGs in cold-responsive hormone signaling pathways. Green boxes show mapped DEGs in phytohormone signaling pathways

We identified 115 DEGs involved in cold-processed hormone signal transduction in Lanjian No. 1 and Lanjian No. 3. These DEGs participate in the signal transduction pathways of auxin, gibberellins (GA), abscisic acid (ABA), cytokinin (CK), ethylene (ET), jasmonic acid (JA), brassinosteroids and salicylic acid (SA) (Fig. 6; Additional file 8 Supplementary Table S5). Most of the known genes in these signaling pathways were identified as DEGs in this study. Differences in the DEG expression patterns were observed between the two cultivars, indicating that the regulation of these common DEGs is related to different levels of cold tolerance. We also comparatively analyzed the expression of DEGs of intermediates in the auxin and ABA pathways (Fig. 6), and the expression of these DEGs was significantly regulated by cold stress in the two cultivars, especially after CS, CA and FA treatments. In addition, the expression patterns of eight key regulatory genes in the auxin pathway were selected and validated using qRT–PCR (Additional file 5 Supplementary Table S2, Additional file 2 Additional file 2 Fig. S2).

### Coexpression network analysis

**Fig. 7:**
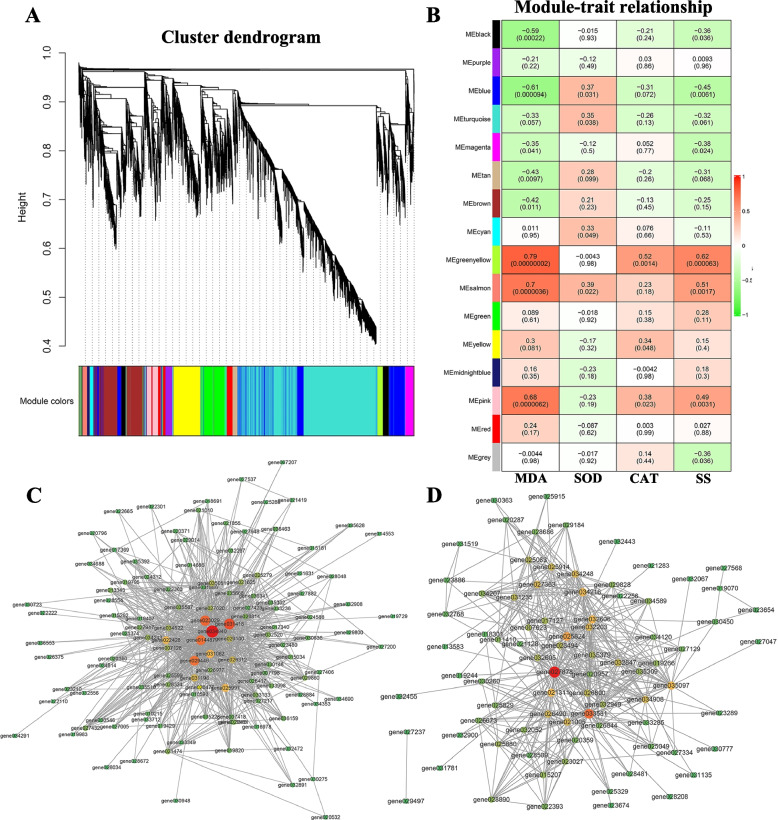
Weighted gene coexpression network analysis (WGCNA) associated with cold acclimation. **A** Coexpression module clustering dendrogram. It mainly consists of 16 modules, which are represented by different colors. **B** The correlation heatmap between modules and physiological indicators. Each row corresponds to a module represented by a different color. Each column corresponds to a physiological indicator. Correlation and *p* values are displayed within each cell. Visualization of green–yellow modules (**C**) and salmon (**D**) modules of key coexpression networks

Weighted correlation network analysis (WGCNA) was performed to investigate the coexpression network of unigenes and to further understand changes in redox and osmoregulation pathways during the cold acclimation of common vetch seedlings. Sixteen coexpression modules were identified based on their similar expression patterns (Fig. 7A, Additional file 9 Supplementary Table S6). Investigations of the relationships between these physiological indexes (MDA, SOD, CAT and SS) and module eigengenes revealed correlation coefficients ranging from − 0.61 to 0.79 for MDA, from − 0.23 to 0.39 for SOD, from − 0.31 to 0.52 for CAT and from − 0.45 to 0.62 for SS (Fig. 7B). At the *P* < 0.05 level, eight modules were associated with the MDA content, four modules with the SOD content, three modules with the CAT content and seven modules with the SS content. The eigengenes in the green–yellow and salmon modules showed significant positive correlations (*P* < 0.01) with MDA, CAT and SS contents, suggesting that these eigengenes might be highly relevant to the cold acclimation of common vetch (Fig. 7B). Furthermore, the first three hub genes in the green–yellow module were annotated from *Medicago truncatula*: a lipid transfer protein, a basic blue-like protein and a 3-ketoacyl-CoA synthase-like protein. The first three hub genes in the salmon module contained one structure-specific endonuclease subunit slx1 from *Cicer arietinum*, one putative thioredoxin m2 (chloroplast) from *Pisum sativum* and one uncharacterized protein LOC101504387 isoform X1 from *C. arietinum* (Fig. 7C, D; Additional file 9 Supplementary Table S6).

### Determination of exogenous IAA and DEG functions

**Fig. 8:**
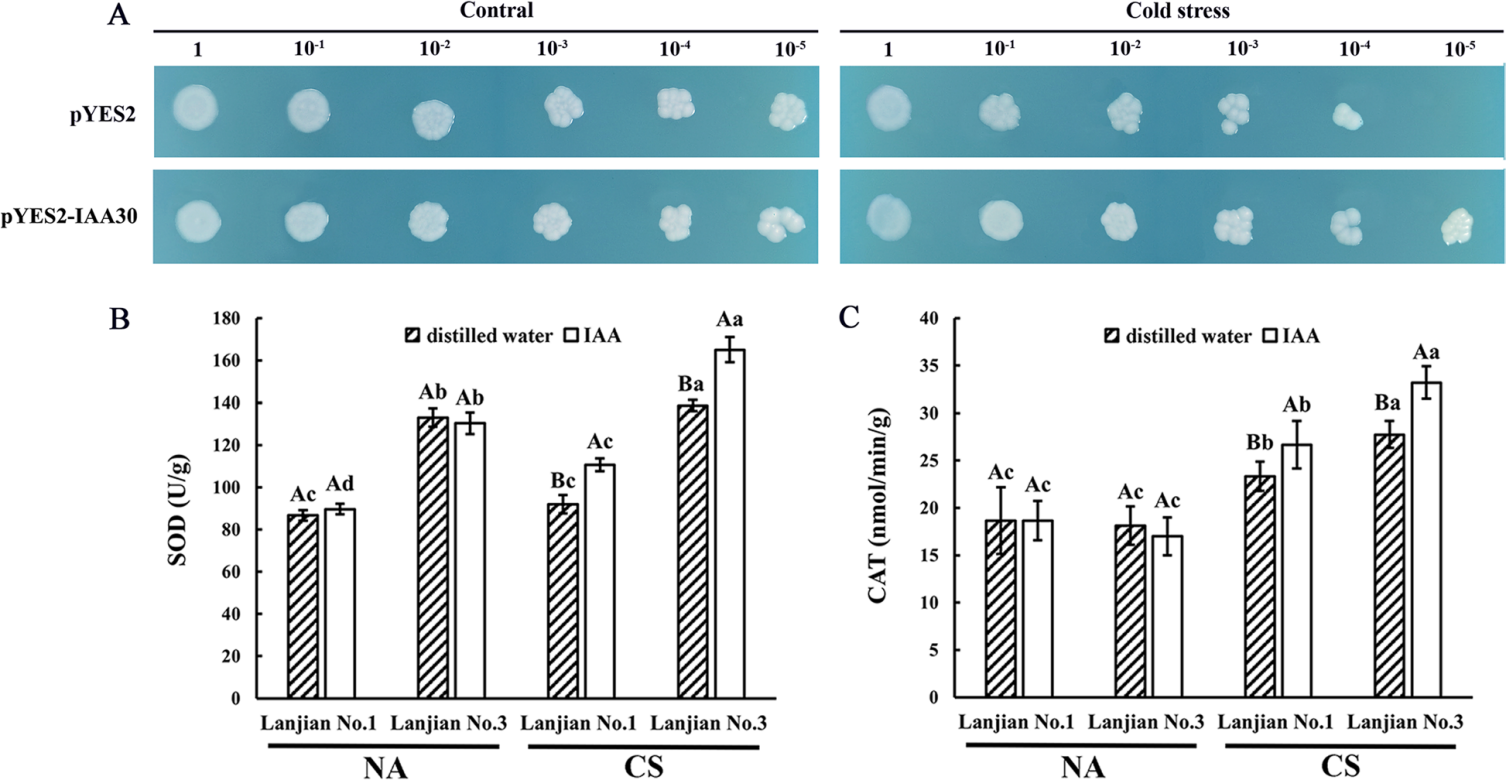
Determination of exogenous IAA and DEG functions. **A** Phenotypic growth assay of transgenic INVSc1 cells under cold stress. Aliquots of serially diluted (1, 10^− 1^, 10^− 2^, 10^− 3^, 10^− 4^, and 10^− 5^) cultures on SC-Ura medium after cold treatment with the pYES2 empty vector and pYES2-IAA30. **B** and **C** Dynamic physiological changes after addition of exogenous IAA. Different capital letters indicate significant differences within the same treatment of the same variety. Different lowercase letters indicate significant differences among the different treatments of the same variety. *p* < 0.05

One DEG (IAA30: c83081.graph_c0) was overexpressed in the cold-sensitive *S. cerevisiae* yeast strain INVSc1, and its growth characteristics were observed to further study the relationship between the identified DEGs and cold acclimation. The yeast strain INVSc1 used in this study was obtained by mating AFB2- and IAA containing strains with ARF containing strains. No difference in growth rate was observe between yeast transformed with the empty and recombinant plasmids under normal culture conditions at 28 °C (Fig. 8A). After treating the transgenic yeast at 4 °C for 36 h, the cells expressing the recombinant plasmid showed a higher growth rate than the cells expressing the empty vector. After cold stress, cells expressing pYES2-IAA30 were still able to grow at a 10^− 5^ dilution, but pYES2-expressing transgenic yeast cells were rarely able to grow at a 10^− 4^ dilution. Thus, potential candidate genes with cold tolerance can be screened among these DEGs, and the cold tolerance of common vetch can be improved through molecular breeding. To demonstrate the effect of IAA on cold stress, we sprayed exogenous IAA on the leaves of cold-acclimated treatment common vetch seedlings. The results showed that after the application of exogenous IAA, the SOD and POD activities of the two varieties were significantly higher than those of the NA treatments, and Lanjian No.3 was significantly higher than Lanjian No.1 (Fig. 8B, C).

## Discussion

A shortage of high-quality forage grass has been noted in high-elevation and cold areas, and traditional perennial forage grass has difficulty surviving winter in these locations [22]. Therefore, clarifying cold resistance genes for the cultivation of new common vetch varieties and to extend their growth period and obtain higher yields is very important. Researchers have reported the expression of several TFs, genes and metabolites in common vetch in response to cold stress, but these studies have not considered cold acclimation and differences between tolerant varieties [1, 23]. This study conducted a systematic analysis of the transcriptomes of common vetch cultivars with high or low cold tolerance under cold acclimation, which will help to accurately understand the cold tolerance mechanism of common vetch.

### Role of Ca^2+^ signaling in the cold acclimation response

Calcium signaling plays a crucial role in mediating various defense responses in plants exposed to environmental stress [24]. In this study, 57 genes related to Ca^2+^ signaling were detected, of which 40 were DEGs (Additional file 10 Supplementary Table S7). These genes are mainly classified as calcineurin B-like protein (CBL), CBL-interacting protein kinase (CIPK), calcium-dependent protein kinase (CDPK), calmodulin-like protein and calcium-transporting ATPase. Notably, the expression of most of the DEGs identified Lanjian No. 3 was induced to a significantly higher level than those in Lanjian No. 1 when treated with CA, but the expression of most genes was induced to a significantly lower level when plants were treated with FA, consistent with the results reported in *Camellia sinensis* in response to cold acclimation. CBLs sense calcium signals generated by environmental stress and then transmit the information to different CIPKs [25]. In *Arabidopsis*, overexpression of the *AtCIPK21* gene reduces cellular lipid peroxidation and ion leakage and increases antioxidant enzyme activity, thereby enhancing cold tolerance [26]. Previous studies have shown that different CBL/CIPK combinations may be involved in modulating specific Ca^2+^ signaling pathways in response to different stimuli [25]. Among the 40 DEGs described above, 5 were identified as CBLs and 4 as CIPKs. CBLs were generally downregulated in response to each treatment, but the expression of 4 of them was significantly upregulated in Lanjian No. 3 compared with Lanjian No. 1 after the CA and FA treatments. Interestingly, the expression profiles of CIPKs were similar to those of CBLs, but CA-treated Lanjian No. 3 showed significantly greater expression of these genes than Lanjian No. 1. Although the difference between the results obtained after CA and NA treatment in Lanjian No. 3 was not significant, the change was significantly higher than that in other treatment groups. An analysis of calmodulin-like protein and calcium-transporting ATPase also showed that the DEG expression level in Lanjian No. 3 was significantly higher than that in Lanjian No. 1 after CA treatment. Therefore, we speculated that components of Ca^2+^ signaling pathways, such as CBL/CIPK, in tolerant cultivars are expressed earlier than those in sensitive cultivars (from NA treatment to CA treatment), such that they respond to cold signals more quickly and prepare for low temperature faster.

### Redox regulation of the cold acclimation response

Among different cold stress regulation strategies, the redox regulatory system is considered one of the main mechanisms, preventing ROS-induced damage to plant cells []. Various enzymatic and nonenzymatic antioxidant mechanisms are used by plants to maintain redox homeostasis. Among them, the main enzymatic antioxidants include peroxidase (POD), CAT, SOD, respiratory burst oxidase (RBOH), alpha-dioxygenase (α-DOX) and peroxiredoxin (Prx) [29]. As shown in the present study, cold acclimation activated the antioxidant defense system of common vetch, and the GO category “antioxidant activity” was significantly enriched in both cultivars (Fig. 4A). Among them, POD contained 31 DEGs. After CA and FA, 17 DEGs were upregulated in Lanjian No. 3, but only 9 were upregulated in Lanjian No. 1 (Additional file 11 Supplementary Table S8). The CAT and α-DOX results were similar to those of POD. For Prx, FA treatment of Lanjian No. 3 resulted in significantly higher expression than in Lanjian No. 1. For SOD and RBOH, most of the DEGs identified in Lanjian No. 1 were expressed at significantly higher levels than in Lanjian No. 3. Based on their different expression patterns and functions in redox regulation, these genes may play different roles in the response to cold acclimation and antioxidant accumulation. Moreover, they may adopt different strategies to cope with cold acclimation, thereby conferring Lanjian No. 3 with greater cold tolerance.

The mitogen-activated protein kinase (MAPK) cascade is a signaling module in eukaryotes that converts external environmental signals into molecular and cellular responses, including MAPK, MAPK kinase (MAPKK) and MAPKK kinase (MAPKKK), which are located downstream of second messengers and hormones [30, 31]. Constitutive activation of the MKK4/5-MPK3/6 cascade negatively regulates the cold response; constitutive activation of the MEKK1-MKK2-MPK4 pathway inhibits the activity of MPK3 and MPK6 and positively regulates the cold response [32]. In the present study, 21 DEGs were identified as MAPKs, 20 as MAPKKs, and 8 as MAPKKKs (Additional file 12 Supplementary Table S9). Three MAPK-related DEGs were upregulated after CA or FA in Lanjian Nos. 1 and 10 DEGs were upregulated after CA or FA in Lanjian No. 3, and the expression levels of 9 DEGs were significantly higher in Lanjian No. 1 after CA. MAPKK and MAPKKK expression profiles were similar to those of MAPKs. Research has reported that MPK3, MPK4, and MPK6 are rapidly activated after exposure to cold stress, and *Arabidopsis mpk3* and *mpk6* mutants exhibit enhanced frost resistance [32]. In the present study, a DEG (c84608.graph_c0) annotated as MPK3 was also upregulated after CS, CA and FA treatments in both cultivars, and it was expressed at significantly higher levels in the Lanjian No. 3 after CS and CA treatments than in Lanjian No. 1. Based on these results, cold treatment at different temperatures rapidly induces the MAP kinase signaling cascade and that different common vetch varieties may activate different regulatory mechanisms to respond accordingly.

### Role of the circadian clock in the cold acclimation response

The circadian clock of plants provides them with a competitive advantage in their living environment by regulating their physiological and metabolic rhythms. Circadian clocks are composed of transcription-translation feedback loops that are influenced by signals such as external temperature and light to adjust biological rhythms to match the environment [33, 34]. In this study, 25 DEGs were enriched in the KEGG pathway “circadian rhythm-plant”; in the CA treatment group, Lanjian No. 3 expressed 16 DEGs at significantly higher levels than those detected in Lanjian No. 1; in the FA treatment group, the expression levels of only 12 DEGs were significantly higher than those in Lanjian No. 1 (Additional file 13 Supplementary Table S10). Among these upregulated DEGs, 1 MYB-domain TF and 7 pseudoresponse regulator (PRR) proteins were identified. PRR is a clock component associated with cold responses. PRR5, PRR7, and PRR9 are involved in a mechanism that predicts circadian cold stress, and the *Arabidopsis prr9–11 prr7–10 prr5–10* triple mutant mediates stress responses by inducing the expression of genes such as DREB1/CBF for cold tolerance [35]. Seven DEGs were annotated as PRRs. Except for one DEG (c93981.graph_c0, PRR1) that was downregulated after CA in Lanjian No. 1, these genes were upregulated after CS, CA and FA in both cultivars. In addition, we detected 2 DREB1s that were upregulated in Lanjian No. 3 after both CA and FA treatments, further suggesting that the clock mechanism affects the cold stress response.

### Hormonal control of cold acclimation responses

The process of cold acclimation may be dynamically regulated by plant hormones, which are primarily influenced by crosstalk with signaling cascades that propagate plant responses to external environmental stimuli such as temperature [36, 37]. Plant hormones are divided into two categories according to their responses to external stress conditions. The first category is “positive growth regulators,” which include hormones such as auxin, gibberellin (GA), brassinosteroids (BR) and cytokinin (CK). The second group is “stress hormones”, which include hormones such as abscisic acid (ABA), salicylic acid (SA), ethylene (ET) and jasmonates (JA) [38]. Cold stress reversibly influences auxin response pathways, affecting growth and development by inhibiting auxin transport [39]. In the present study, 55 auxin-responsive DEGs were detected (Fig. 6; Additional file 8 Supplementary Table S5). Aux/IAA proteins are a class of short-lived transcriptional regulators that mediate auxin responses through interactions with ARFs [40]. For AUX/IAA, Lanjian No. 1 showed 10 DEGs that were upregulated after CA and FA. In Lanjian No. 3, 16 DEGs were upregulated after CA and FA treatments, and 16 DEGs were expressed at significantly higher levels in this cultivar after CA treatment than in Lanjian No. 1. Except for GH3, the expression of AUX1, ARF and small auxin upregulated RNA (SAUR) was consistent with the expression profile of AUX/IAA. Downregulation of SAUR genes confers tolerance to various abiotic stresses in plants [41]. *OsGH3–2* is a member of the GH3 family and encodes an enzyme that catalyzes the attachment of indoleacetic acid (IAA) to amino acids [42]. Further studies showed that the IAA content and cellular oxidative damage are associated with cold tolerance in *OsGH3–2*-overexpressing rice, with a reduction in IAA content resulting in a reduction in ABA content [43]. In addition, changes in the expression of IAA catabolism-related genes, such as the GH3 family, were opposite to the changes in the expression of IAA biosynthesis genes observed in plants under abiotic stress [40]. In the current study, 4 and 5 DEGs related to GH3 were downregulated after CA and FA treatments in the two cultivars, respectively, and 3 DEGs identified in Lanjian No. 3 after CA treatment were expressed at significantly lower levels than in Lanjian No. 1. Thus, Lanjian No. 3 increased the level of IAA through the interaction of the highly expressed transcriptional regulator Aux/IAA with ARF, while the low expression of GH3 decreased the decomposition of IAA, thus endowing it with stronger cold tolerance. Although we overexpressed an IAA gene in *S. cerevisiae* and showed that it conferred stronger cold tolerance, the mechanism of cold domestication of common vetch related to IAA still remains to be further determined.

GAs play a central role in conferring the ability to adapt to changing external environments in plants [37]. GA metabolism and signaling are activated in response to cold stress, and studies have shown that CBFs are involved in this process. Low temperature-induced CBF1 expression inhibits growth by promoting DELLA accumulation, while GA stimulates DELLA degradation [44]. In addition, growth is inhibited when overexpressed by cold-induced *ZAT10*, *ZAT12* and other primary responses and by TFs that control non-CBF regulons [37]. Here, the expression profiles of DEGs enriched in the GA signaling pathway differed between the two cultivars (Additional file 8 Supplementary Table S5). Among them, 3 DELLAs were upregulated in both cultivars, 4 GID1 s were only upregulated in Lanjian No. 3, and 1 DEG identified as a TF was upregulated in both cultivars, while the remaining DEGs were downregulated. Based on this result, the growth of common vetch might be inhibited by DELLA accumulation after cold acclimation.

CK promotes the differentiation and growth of plant tissues and exerts a synergistic effect with auxin [45]. Studies have shown that cold induces ARR expression through AHK2 and AHK3 proteins but does not alter cytokinin levels. Furthermore, *AHK2* and *AHK3* and cold-inducible A-type *ARRs* negatively regulate cold-responsive signaling by inhibiting ABA, independent of the cold acclimation pathway [46]. In this study, 8 DEGs were detected in the CK signaling pathway, including 1 AHP, 2 CRE1 and 5 B-ARRs (Additional file 8 Supplementary Table S5). Among them, only 1 CRE1 was upregulated in Lanjian No. 3, and 2 B-ARRs were upregulated in Lanjian No. 1. Thus, the acquisition of cold tolerance in Lanjian No. 3 may be related to the low expression of ARRs.

ABA is an isoprenoid hormone that plays an important role in responding to abiotic stresses such as cold stress [37]. Exogenous application of ABA improves the frost resistance of *Arabidopsis*, and ABA mutants also show better cold tolerance [47]. ABA induces the expression of MYB96 and controls the transcription of CBF [48]. Twenty-nine ABA-related DEGs were detected in this study (Fig. 6, Additional file 8 Supplementary Table S5). Among them, 9 PP2Cs were all upregulated in Lanjian No. 3 after CA treatment, and 6 PP2Cs were upregulated in Lanjian No. 1 after CA treatment. However, PYR/PYL were downregulated in both varieties. Previous studies have found that PYR/PYLs are a family of ABA receptors and that ABA and PYR/PYLs inhibit PP2Cs [49]. *MtPP2C46*, *MtPP2C47* and *MtPP2C72* expression were induced by cold, drought and ABA and played an important role in the cold response [50]. As shown in the present study, cold acclimation of common vetch may promote PP2C expression through the downregulation of PYR/PYLs in the ABA regulatory pathway, thereby conferring cold tolerance.

ET and JA often act synergistically and play an important role in regulating plant tolerance to abiotic stresses. Under cold stress, ET and JA constitute a regulatory network that differentially regulates the CBF pathway. Complex regulation is accomplished through differential expression of components in their respective pathways, such as EIN2, EIN3, ERF, JAZ, and MYC2 [51]. Evidence has shown that the ET signaling pathway may inhibit the CBF/DREB1 pathway in soybean (*Glycine max* L.) through the action of EIN3 [52]. Overexpression of *MdERF1B* significantly improves the cold tolerance of apple (*Malus* × *domestica* Borkh.) and *Arabidopsis* seedlings. Moreover, *MdCIbHLH1* enhances *MdERF1B* binding to target gene promoters and subsequent transcriptional activation. Following cold stress, an increase in the JA content triggers COI1-mediated JAZ degradation, which releases INDUCER OF CBF EXPRESSION (ICE) from its repressed state. Then, ICE1 and ICE2 bind to the *DRE/CRT* box elements in the *ICE*-regulon promoter to activate CBFs. In addition, bHLH, acting downstream of JAZ, is involved in regulating the cold response [51]. In this study, 14 DEGs were identified in the ET pathway. Among them, except for 1 EIN3, which was upregulated in the CA treatment of Lanjian No. 3, the remaining EIN3s were downregulated in the CS, CA and FA treatment groups in both varieties. Eleven DEGs were identified in the JA pathway, including one COI1 and six JAZs (Additional file 6 Supplementary Table S3). For JAZ, 5 and 4 DEGs in Lanjian No. 1 and Lanjian No. 3, respectively, were downregulated in all treatment groups. Therefore, ET, JA, and the cold pathway share several common components that induce the downregulation of EIN3 and JAZ through cold acclimation, attenuate the inhibition of the CBF pathway, and enhance cold tolerance in common vetch.

### TFs involved in cold acclimation responses

Numerous TF families play important regulatory roles in response to cold stress [53]. The AP2/ERF TF family is one of the most important TF families associated with cold stress, and it is interrelated with other TF families, such as MYB, bHLH, C2H2, and WRKY, to enhance cold stress tolerance [54]. In the present study, 49 AP2-related DEGs were monitored, and 8 and 15 were upregulated in all Lanjian No. 1 and Lanjian No. 3 treatment groups, respectively (Additional file 14 Supplementary Table S11). The number of upregulated DEGs identified in Lanjian No. 1 increased from 11 to 20 in response to the CS, CA and FA treatments. Cold acclimation enhanced the cold tolerance of Lanjian No. 1. Studies have shown that plant MYB TFs regulate plant homeostasis in response to various abiotic stresses [55]. For example, *OsMYB2* enhances the tolerance of rice to salt, cold and dehydration stresses [56]. Eighty-six MYB-related DEGs were detected, with 17 and 22 upregulated in all treatment groups in Lanjian No. 1 and Lanjian No. 3, respectively (Additional file 14 Supplementary Table S11). The CA treatment increased the number of upregulated DEGs in the two cultivars, and the expression levels of 51 DEGs in Lanjian No. 3 were significantly higher than those in Lanjian No. 1. This finding is consistent with the results of previous studies. In this study, 63 bHLH-related DEGs were detected, and after cold acclimation, 37 DEGs in Lanjian No. 3 were present at significantly higher levels than in Lanjian No. 1 (Additional file 14 Supplementary Table S11). *VabHLH1* and *VvbHLH1* in *Vitis vinifera* positively regulate the response to cold stress and enhance the cold stress tolerance of plants by regulating the COR expression level [57]. We also detected 27 and 33 WRKY- and C2H2-related DEGs, and after cold acclimation, the levels of 21 and 16 DEGs in Lanjian No. 3 were significantly higher than those in Lanjian No. 1, respectively (Additional file 14 Supplementary Table S11). The expression of the *BcWRKY46* gene in *Brassica campestris* is substantially induced by cold stress and ABA and activated related genes in the ABA signaling pathway, conferring low-temperature tolerance [58]. Soybean cold-inducible C2H2 TF (*SCOF-1*) may play a role in low temperature tolerance, and low temperature and ABA induce the expression of *SCOF-1* [59]. According to these results, TFs are collectively involved in multiple regulatory processes in the common vetch cold-responsive regulatory network.

## Conclusions

The present study provides a comparative transcriptomic analysis that is the first to reveal similar and distinct molecular mechanisms of common vetch cultivars with different cold tolerances under cold acclimation conditions. After the cold signal is sensed, it is first transduced through Ca^2+^ and hormone signaling to activate downstream TFs. TFs are subsequently involved in regulating the expression of related *COR* genes, triggering corresponding physiological and biochemical reactions such as redox reactions and conferring cold tolerance to common vetch (Additional file 2 Fig. S2). The comprehensive analysis revealed the differential response and transcriptional differentiation of two common vetch varieties to cold signals, which will help to reveal the underlying mechanism of cold domestication and provide support for further variety improvement.

## Materials and methods

### Plant materials and treatmentss

Seeds of common vetch cultivars Lanjian No. 1 (late-maturity cold-sensitive) and Lanjian No. 3 (early-maturity cold-tolerant) were sterilized with 1.0% (v/v) sodium hypochlorite for 5 minutes, rinsed with distilled water 5 times, and then placed in the dark at 25 °C and allowed to germinate for 4 days (Additional file 4 Supplementary Table S1). Seeds with consistent germination were transferred to an incubator (25 °C, 16 h/8 h light/dark and 50% relative humidity) for cultivation using 1/2 MS medium (pH = 6.5). After 7 days, the plants were subjected to cold acclimation and deacclimation. Untreated plants were used as nondomesticated controls (NA). The cold acclimation treatments listed below were used. For CS, after 7 days of normal culture, Lanjian No. 1 and Lanjian No. 3 were placed at 4 °C for 6 h. For CA, Lanjian No. 1 and Lanjian No. 3 were placed at low temperature (10 °C) for 5 days after normal culture. Then, the samples were incubated at 4 °C for 5 days for FA. Finally, the plants were placed at room temperature (25 °C) for 5 days for DA. All treatments were performed on a 16-h/8-h (light/dark) photoperiod. After each treatment, the second and third leaves from the top down were collected, immediately frozen in liquid nitrogen, and stored at − 80 °C until use. Three biological replicates from each treatment group were used.

### Measurements of physiological indices

Changes in MDA [1], SOD [60], CAT [60] and soluble sugar contents [61] were detected. These parameters were detected using corresponding kits from Beijing Solarbio Science and Technology Co., Ltd. (Solarbio, Beijing, China).

The chlorophyll fluorescence (Fv/Fm) value was measured using a previously described method [62]. Seven-day-old Lanjian No. 1 and Lanjian No. 3 seedlings were placed at 4 °C for 12 h, and Fv/Fm images of NA or FA leaves were obtained using an Imaging-Pam chlorophyll fluorescence imager (Walz, Germany).

To demonstrate the effect of IAA on cold stress, exogenous application of IAA (100 mg L^− 1^, ICN, Bonn, Germany) and distilled water (0 mg L^− 1^) was sprayed on the leaves of seedlings grown 7 days after germination. Plant leaves were collected for SOD and CAT activity measurements after CS treatments [61].

### RNA extraction and sequencing

RNA samples were extracted from 3 biological replicates of the Lanjian No. 1 and Lanjian No. 3 seedlings from the control and treatment groups. RNA extraction was performed using a previously described method [1]. One percent agarose gels and an Agilent 2100 Bioanalyzer (Agilent Technologies, USA) were used to evaluate the quality and quantity of RNA, respectively. The RNA library was constructed using previously described methods [17]. The 36 cDNA libraries were sequenced with a read length of 150 bp using the Illumina HiSeq 2000 platform with a paired-end module at Biomarker Co. Ltd. (BMKcloud, Beijing, China).

### Transcript assembly and functional annotation

The FASTX toolkit was used to clean the raw sequencing reads [17]. Bowtie2 software was used to map the clean reads onto full-length transcripts. The Trinity and TGICL programs were used to assemble unigenes [63], and all unigenes were subjected to BLASTx for annotation and alignment, including the NCBI nonredundant protein sequence (Nr), GO, Protein Orthologous Group (COG), KEGG [64] and Swiss-Prot databases.

### DEG analysis

DEGs (transcript abundance ≥2, Wald test, *P* < 0.05) were analyzed using the MARS method with the DEGseq 2010 R package. The method reported by Benjamini and Hochberg was applied to correct *P* values, and the corrected *P* values and FDR (false discovery rate) were used to reduce false positives [17]. The GO (version 1.18.0) enrichment analysis was performed using topGO software and agriGO; KOBAS 2.0 was used for the KEGG pathway enrichment analysis [2].

### qRT–PCR validation

A portion of the total RNA extracted from the 36 samples used for the RNA-Seq analysis was employed for the qRT–PCR analysis. The method was the same as the procedure described in a previous report [65]. The primers and internal reference genes for qRT–PCR are described in Additional file 5 Supplementary Table S2.

### WGCNA and gene network visualization

FPKM values for all unigenes and physiological parameters from the same samples were used for WGCNA using the method described in a previous report [66]. Both network construction and coexpression modules were generated using an automatic one-step method with default parameters [67]. The obtained module eigengene values were used to determine the association of different modules with physiological parameters related to the cold acclimation response for each sample. Physiological parameters and highly correlated modules were visualized using Cytoscape.

### Cold tolerance tests in transgenic yeast

The IAA library was cloned into *TRP1* integration vector and transformed into W814-29B *MATa* strains (YKL381) containing pGP5G-AFB2 [AFB2 integrated at *LEU2*]. ARFs were amplified from Arabidopsis cDNA and subcloned into the Gateway pDONR221 plasmid using a standard Gateway BP reaction (BP Clonase II; Life Technologies). Finally, Strains containing AFB2- and IAA were mated to strains containing ARF to generate the yeast strain INVSc1 used in this study. The full-length cDNA sequence was obtained using gene-specific primers, as shown in Additional file 6 Supplementary Table S3. The detailed method was performed as previously described [1, 68]. pYES2 was used as the expression vector (Invitrogen, Carlsbad, USA) in the cold-sensitive yeast strain INVSc1. The cold stress assessment was conducted in SC-Ura medium, and the yeast cells were grown in a shaker at − 4 °C for 36 h. Serial dilutions were spotted onto SC-Ura agar plates and incubated at 28 °C for 72 h; the experiment was repeated three times.

## Supplementary Information

**Additional file 1: Fig. S1.** The qRT-PCR confirmation of eight of the key regulatory genes in auxin signaling pathway. The Y-axis on the left side of each graph represents the expression level (FPKM) of RNA-seq, and the Y-axis on the right side represents the relative expression of qRT–PCR, * *p* < 0.05.

**Additional file 2:** **Fig. S2.** Models describing the regulatory network involved in the acquisition of cold tolerance in common vetch.

**Additional file 3:** Additional file

**Additional file 4:** **Table S1.** Days to 50% flowering, maturity, stem thickness, crop height, dry matter and seed yield of different cultivars

**Additional file 5:** **Table S2.** Primers used for the qRT–PCR analysis

**Additional file 6:** **Table S3.** Primers used to verify the expression of cold tolerance-related genes

**Additional file 7:** **Table S4.** Summary of Illumina sequencing data and assembled results

**Additional file 8:** **Table S5.** Expression of genes related to hormonal signaling in common vetch under cold acclimation conditions

**Additional file 9:** **Table S6.** WGCNA data

**Additional file 10:** **Table S7.** Expression of genes involved in Ca^2+^ signaling in common vetch under cold acclimation conditions

**Additional file 11:** **Table S8.** Expression of genes related to redox processes in common vetch under cold acclimation conditions

**Additional file 12:** **Table S9.** Expression of DEGs related to the MAP kinase cascade in common vetch under cold acclimation conditions

**Additional file 13:** **Table S10.** Expression of DEGs related to plant circadian rhythms in common vetch under cold acclimation conditions

**Additional file 14:** **Table S11.** Expression of genes related to TFs in common vetch under cold acclimation conditions

## Data Availability

The materials of this study were bred by Zhibiao Nan and Bin Nie of Lanzhou University, and the common vetch seeds were obtained from Bin Nie and used with permission. Correspondence and requests for materials should be addressed to Rui Dong (rdong@gzu.edu.cn). The raw sequencing data have submitted to the NCBI SRA database (PRJNA820149).
