# Supplementary figures and images for: A comparative transcriptomic analysis reveals a coordinated mechanism activated in response to cold acclimation in common vetch (Vicia sativa L.)
Source: BMC Genomics. 2022 Dec 8;23:814. doi: 10.1186/s12864-022-09039-w (PMC9733113; doi:10.1186/s12864-022-09039-w)

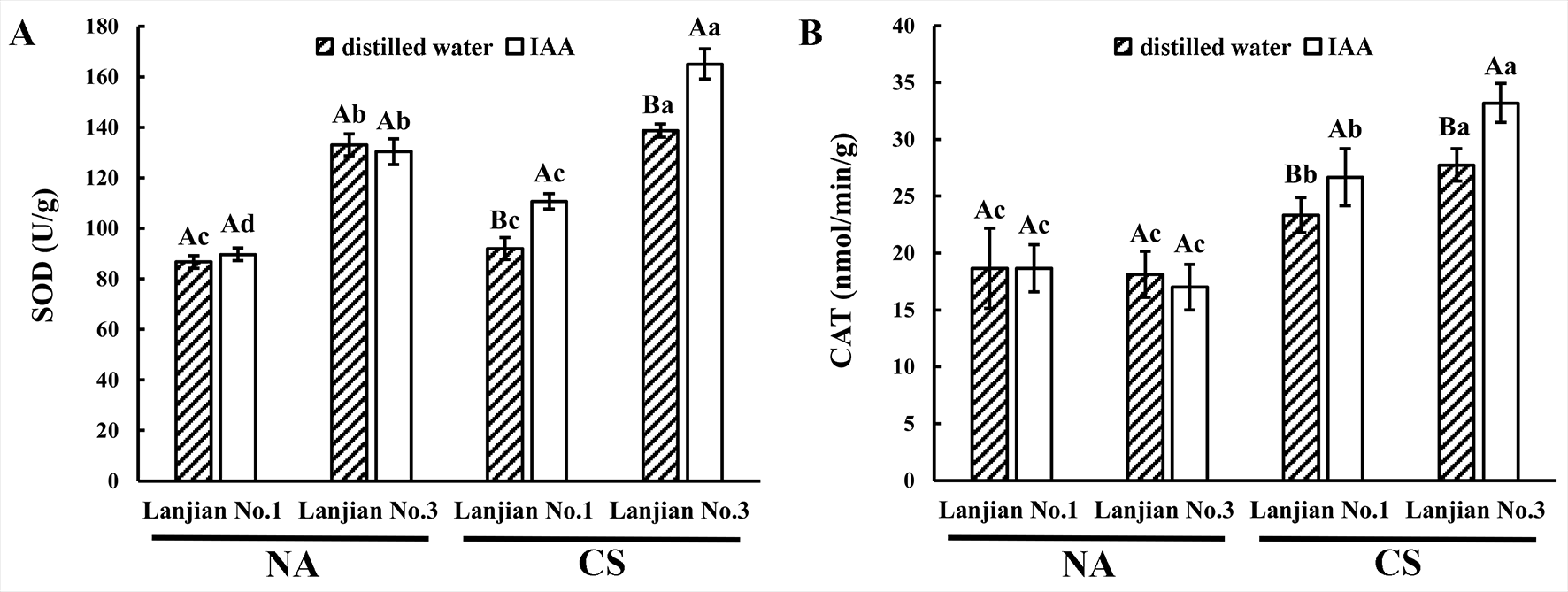

Supplement: Supplementary file 1 — Additional file 1: Fig. S1. The qRT-PCR confirmation of eight of the key regulatory genes in auxin signaling pathway. The Y-axis on the left side of each graph represents the expression level (FPKM) of RNA-seq, and the Y-axis on the right side represents the relative expression of qRT–PCR, * p < 0.05. [file 12864_2022_9039_MOESM1_ESM.tif]

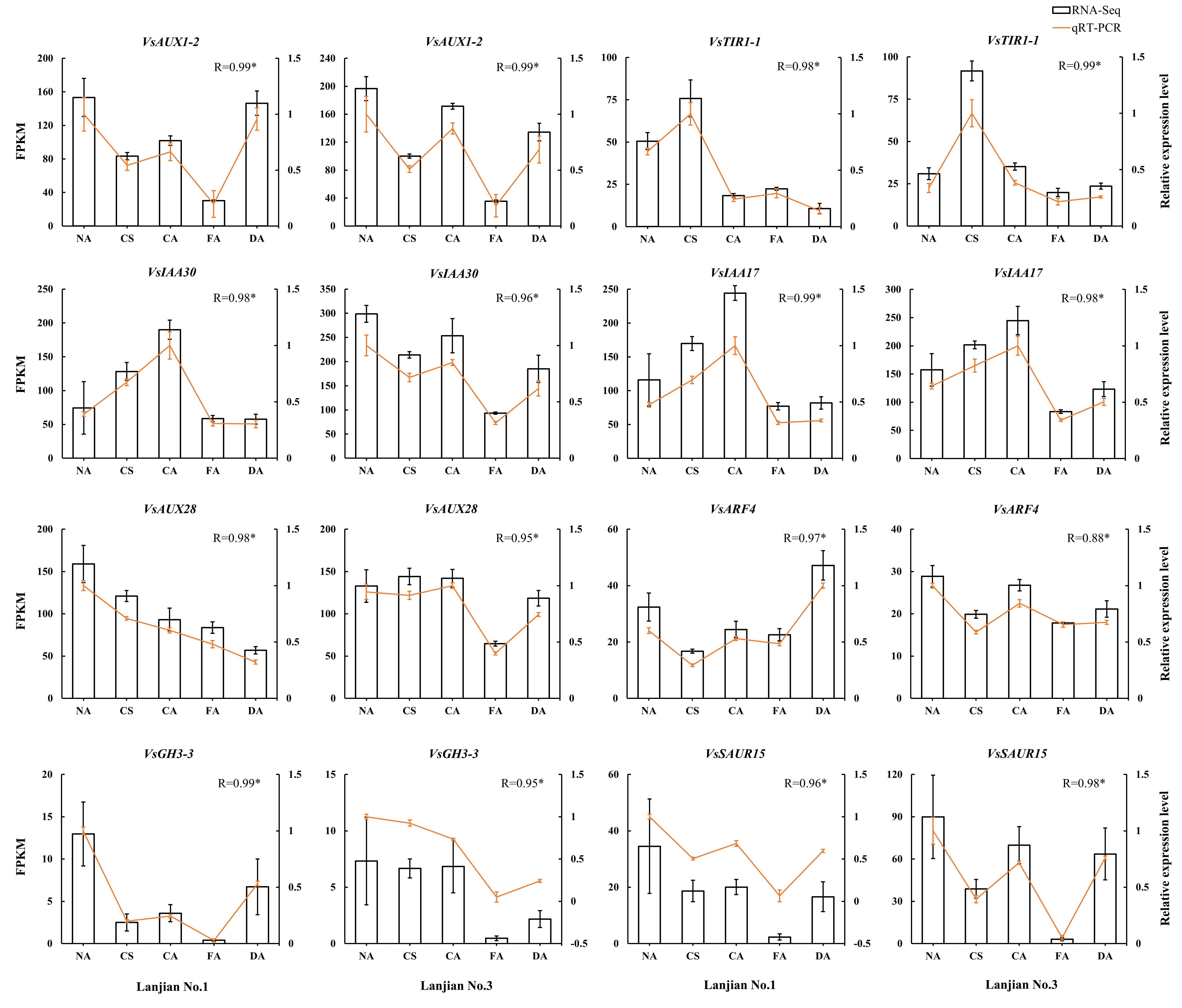

Supplement: Supplementary file 2 — Additional file 2: Fig. S2. Models describing the regulatory network involved in the acquisition of cold tolerance in common vetch. [file 12864_2022_9039_MOESM2_ESM.tif]

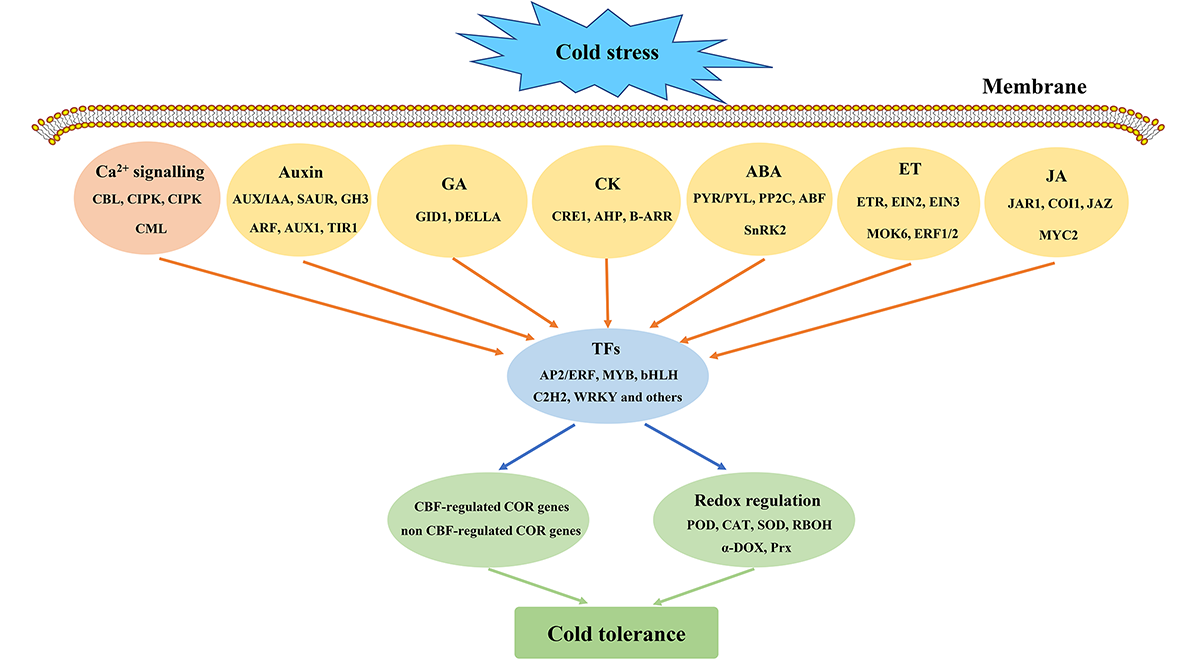

Supplement: Supplementary file 3 — Additional file 3: Additional file [file 12864_2022_9039_MOESM3_ESM.tif]
